# Dynamic ion-buffering gradient bilayer anode realizes 200 Wh kg^−1^ dendrite-free sodium battery

**DOI:** 10.1093/nsr/nwaf427

**Published:** 2025-09-30

**Authors:** Siyang Ye, Shuanghui Han, Fei Tian, Danni Lei, Chengxin Wang

**Affiliations:** State Key Laboratory of Optoelectronic Materials and Technologies, School of Materials Science and Engineering, Sun Yat-sen University, Guangzhou 510275, China; State Key Laboratory of Optoelectronic Materials and Technologies, School of Materials Science and Engineering, Sun Yat-sen University, Guangzhou 510275, China; State Key Laboratory of Optoelectronic Materials and Technologies, School of Materials Science and Engineering, Sun Yat-sen University, Guangzhou 510275, China; State Key Laboratory of Optoelectronic Materials and Technologies, School of Materials Science and Engineering, Sun Yat-sen University, Guangzhou 510275, China; State Key Laboratory of Optoelectronic Materials and Technologies, School of Materials Science and Engineering, Sun Yat-sen University, Guangzhou 510275, China

**Keywords:** sodium battery, anode design, ion-buffering interlayer, dendrite free, Na compensation

## Abstract

The development of sodium batteries is hindered by dendrite growth and sodium ion loss in conventional anodes under practical operating conditions. We engineer a gradient sodium–tin alloy/sodium bilayer anode through *in situ* chemical displacement. The upper gradient alloy phase serves as an ion-buffering interlayer that synergistically regulates thermodynamic driving forces and ion-transfer kinetics to achieve dendrite-free morphology. The underlying metallic sodium layer functions as an ion reservoir dynamically compensating for sodium ions to maintain the structural stability of the gradient phase and mitigate the consumption of sodium during long-term cycling. The resulting symmetric cells demonstrate ultralong cyclability exceeding 7000 h at a current density of 3 mA cm^−2^. Encouragingly, when paired with a high-loading Na_3_V_2_(PO_4_)_3_ cathode (30 mg cm^−2^), the full cell cycles stably for nearly 1000 cycles and delivers an unprecedented energy density of 200 Wh kg^−1^. This work establishes a materials design paradigm to address fundamental challenges in post-lithium battery systems.

## Teaser

Gradient sodiumtin alloy/sodium bilayer anode buffers ion flux and compensates sodium loss, suppressing dendrites and preserving structural stability. This unique architecture design endows sodium batteries with high-energy-density and ultralong cycling life.

## INTRODUCTION

The development of sustainable energy storage systems demands cost-effective and high-performance battery technologies [1]. Na-based batteries have emerged as promising alternatives to conventional lithium-ion batteries in the renewable energy sector, primarily due to the greater natural abundance and lower cost of Na [2]. However, the realization of high-energy-density Na batteries faces fundamental challenges at the anode, where three critical requirements converge: high capacity; Na compensation capability; and dendrite suppression.

Current anode materials present critical limitations through distinct failure mechanisms. Hard carbon anodes (350−400 mAh g^−1^) suffer from defect-induced electrolyte decomposition and Na^+^ consumption, as well as Na plating during fast charging [3–5]. Alloy materials like Sn (847 mAh g^−1^) eliminate plating risks but lack Na compensation capability while enduring 300%–400% volume changes [6,7]. Metallic Na anodes (1166 mAh g^−1^) theoretically maximize energy density [2,8,9] but face dendrite proliferation through unstable solid electrolyte interphase (SEI) evolution [10,11]. These fundamental limitations persist despite composite strategies combining Na metal with sodiophilic substrates or alloy phases [7,12–17], approaches that partially address volume changes but fail to modify the intrinsic Na deposition mechanism.

Recent composite designs reveal three unresolved challenges: (i) inadequate mechanical resilience to accommodate >8 μm interfacial fluctuations during cycling; (ii) increased electrolyte decomposition at high-surface-area interfaces; and (iii) limited effectiveness of dispersed alloy phases in guiding Na deposition morphology. Crucially, no existing architecture alters the Na plating and stripping mechanism, thus failing to fundamentally solve the dendrites problem. Therefore, achieving a dendrite-free and Na-compensating anode remains a significant challenge for Na-based batteries under practical harsh conditions (cathode mass loading > 20 mg cm^−2^).

Here, we engineer a gradient Na–Sn/Na bilayer anode (GNS/Na) via *in situ* chemical displacement involving Sn ethoxide reduction and controlled interdiffusion. The compositionally modulated unsaturated Na_x_Sn_y_ alloy (x/y ≤ 15/4) serves as an electrochemical buffer layer, which simultaneously optimizes both the thermodynamics and kinetics of ion diffusion, thereby achieving dendrite-free morphology. Beneath this gradient layer, the bulk Na reservoir ensures both the stability of gradient structure and compensation of Na^+^ depletion through dynamic ion replenishment during extended cycling. Therefore, this work redefines anode design principles. As a result, the symmetric cell achieves an ultralong cycle life of 7000 and 700 h at current densities of 3 and 10 mA cm^−2^, respectively. Notably, when paired with a high-loading Na_3_V_2_(PO_4_)_3_ (NVP) (30 mg cm^−2^) cathode, the full cell cycles stably for nearly 1000 cycles and delivers an unprecedented energy density of 200 Wh kg^−1^. We establish a materials design paradigm that simultaneously addresses capacity, stability and safety–key barriers to sustainable energy storage. This strategy can be extended to post-lithium batteries.

## RESULTS AND DISCUSSION

### Design and potential profiling characterization of gradient electrode

**Figure 1.**
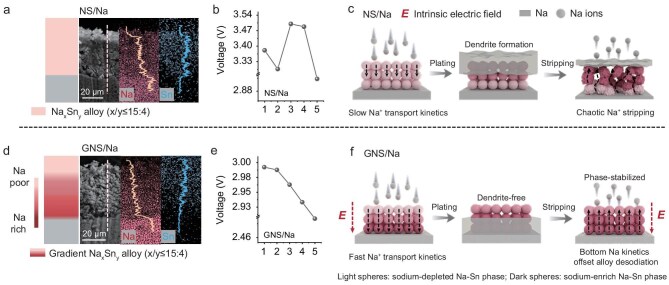
Design and characterization of gradient electrode. (a) Cross-sectional SEM image, the corresponding EDS mapping and the corresponding elemental line scan of NS/Na. (b) The measured potential of NS/Na. (c) Schematic illustration of the NS/Na electrode after Na plating and stripping process. (d) Cross-sectional SEM image, the corresponding EDS mapping and the corresponding elemental line scan of GNS/Na. (e) The measured potential of GNS/Na. (f) Schematic illustration of the GNS/Na electrode after Na plating and stripping process. We prepared cross-sectional samples of NS/Na and GNS/Na, measuring the potential in five 5-μm regions from top to bottom of the modified layer using Kelvin probe force microscopy (KPFM) (Figs S7 and S8).

In order to illustrate the rationality of GNS/Na electrode design, we prepared gradient Na–Sn alloy anode (GNS) and Na–Sn/Na bilayer anode (NS/Na) electrodes, respectively. The GNS was synthesized by combining Na_9_Sn_4_ (unsaturated, higher potential) and Na_15_Sn_4_ (saturated, lower potential) phases [18,19] to investigate the role of intrinsic electric fields in Na⁺ transport dynamics (Figs S1–S6). Although the built-in electric field in GNS effectively modulates Na⁺ transport dynamics, structural degradation occurs during prolonged cycling due to Na consumption and significant volumetric strain induced by the repetitive sodiation/desodiation process. In the NS/Na sample lacking composition (Fig. 1a) and potential (Fig. 1b) gradient design, the inadequate ionic transport kinetics within the alloy phase induce persistent Na accumulation in upper regions. This spatial redistribution generates a reverse electric field that impedes directional ion migration, with such electric field inversion promoting Na dendrite formation on the alloy surface. During subsequent stripping cycles, the irreversible stripping failure of surface Na metal forces Na^+^ extraction from the alloy phase, and thus triggers its mechanical fracture. Concurrently, the Na^+^ released from the underlying metallic Na layer will migrate through the interparticle gaps to replenish the Na loss in the cathode, but they cannot be effectively replenished into the alloy layer to stabilize its structure (Fig. 1c). These findings underscore the necessity of strategically integrating GNS with metallic Na to achieve sustainable battery operation. Consequently, we engineered a GNS/Na composite anode with composite (Fig. 1d) and potential gradients (Fig. 1e), hypothesizing that the upper gradient alloy phase serves as an ion-buffering interlayer that synergistically regulates thermodynamic driving forces and ion-transfer kinetics for dendrite-free deposition. Meanwhile, the underlying metallic Na layer functions as a dynamic ion reservoir, maintaining structural stability of the gradient phase and mitigating Na consumption during long-term cycling (Fig. 1f).

### Characterization of GNS/Na and NS/Na electrode

The preparation process begins with a displacement reaction between Sn(EtO)_2_ and Na metal in cyclohexane solution, producing Sn particles and NaEtO on the Na surface (Equation 1). The resulting electrode is named NS/Na. The NS/Na is then heated to 110°C for 20 min, causing the Na metal at the bottom to melt and diffuse upwards, forming a Na–Sn alloy with varying degrees of sodiation (Equation 2).


(1) \begin{eqnarray*}
2{\mathrm{Na}} + {\mathrm{Sn}}{\left( {{\mathrm{EtO}}} \right)}_2 = {\mathrm{Sn}} + 2{\mathrm{NaEtO}}
\end{eqnarray*}

(2) \begin{eqnarray*}
{\mathrm{xNa}} + {\mathrm{ySn}} = {\mathrm{N}}{{\mathrm{a}}}_{\mathrm{x}}{\mathrm{S}}{{\mathrm{n}}}_{\mathrm{y}}\left( {0 < {\mathrm{x}}/{\mathrm{y}} < 15/4} \right)
\end{eqnarray*}

**Figure 2.**
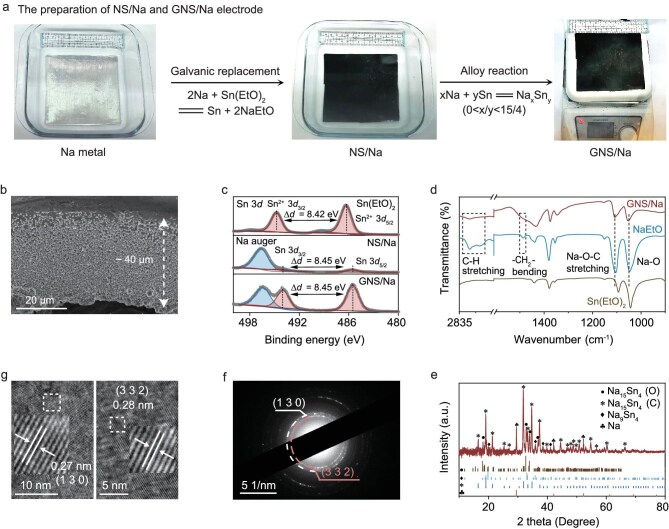
Physicochemical characterizations of gradient Na–Sn alloy/Na composite electrode. (a) The fabrication of NS/Na and GNS/Na electrodes. (b) Cross-sectional SEM image of the modified alloy structure. (c) XPS spectra of Sn 3*d* for Sn(EtO)_2_, NS/Na and GNS/Na. (d) FTIR spectra of NaEtO, Sn(EtO)_2_, and GNS/Na. (e) XRD pattern of the modified layer that scraped off from GNS/Na. Selected-area electron diffraction (f) and HRTEM images (g) of GNS/Na.

The diffusion of Na into Sn follows the principles of Fick’s law, with the driving force being the gradient of the chemical potential. This gradient is governed by both the concentration difference and the energy difference between the Na metal and Sn particles. Na diffuses from the region of higher chemical potential (Na) to the region of lower chemical potential (Sn), in accordance with the thermodynamic principle of minimizing the system’s Gibbs free energy. The resulting electrode is named GNS/Na (Fig. 2a). The thickness of the modified structure is regulated by controlling the time of soaking. The thickness of the modified structure increases progressively with immersion time, reaching approximately 2.6 μm after 10 min, 6.7 μm after 30 min, 10 μm after 50 min, and 40 μm after 3 h (Fig. S9). Notably, a by-product layer of NaEtO, approximately 1.5 μm thick, covers the reduced modified structure (Fig. S10). We optimize the content of the Na–Sn alloy and improve the cycling performance of the GNS/Na electrode; the soaking time and heating temperature are adjusted. As shown in Fig. S11, the Na metal soaked in cyclohexane for 3 h and kept at 110°C for 20 min has the smallest overpotential and the best cycle stability in the range of 0.25–6 mA cm^−2^. Therefore, the most optimal treatment condition to prepare GNS/Na is obtained. Different from the Na–Sn alloy/Na composite anode prepared by calendering a mixture of Na and Sn powders/foils, our gradient alloy phase, approximately 40 μm thick (Fig. 2b), contains a range of unsaturated Na_x_Sn_y_ alloys (x/y ≤ 15/4). Additionally, the by-product NaEtO serves as an electronic insulator and Na^+^ conductor, further mitigating dendrite growth on the surface. The composition of the modified structure was analyzed by X-ray photoelectron spectroscopy (XPS). Figure 2c shows the Sn 3*d* spectra for Sn(EtO)_2_, NS/Na and GNS/Na. The binding energy of Sn 3*d*_3/2_ and Sn 3*d*_5/2_ for Sn(EtO)_2_ is 494.77 and 486.31 eV [20], respectively, while the binding energy of Sn 3*d*_3/2_ and Sn 3*d*_5/2_ for NS/Na and GNS/Na move to 493.96 and 485.51 eV [21], respectively. The decrease in binding energy indicates that Sn^2+^ ions are reduced to metallic Sn. To verify the presence of the NaEtO by-product, Fourier-transform infrared spectroscopy (FTIR) was used to characterize the GNS/Na. The FTIR results for GNS/Na show vibrational peaks consistent with those of commercial NaEtO, with peaks at 1047 and 1105 cm^−1^ corresponding to the stretching of Na–O–Na and Na–O–C bonds, respectively (Fig. 2d). The NaEtO formed in our electrode remains largely insoluble in the electrolyte and the trace amount of dissolved NaEtO would have a positive effect on the overall battery performance (Figs S12 and S13). The modified structure of GNS/Na was scraped off and analyzed by X-ray diffraction (XRD), which revealed that the obtained structure constituted the components of the Na_15_Sn_4_ alloy (cubic and orthorhombic phases) and Na_9_Sn_4_ alloy (Fig. 2e). The structure of the GNS/Na was further characterized by the selected area electron diffraction (SAED) pattern (Fig. 2f) and high-resolution transmission electron microscopy (HRTEM) (Fig. 2g), revealing various grains corresponding to different components. The lattice fringes with a spacing of 0.27 nm match well with the (130) planes of Na_9_Sn_4_, while the lattice fringes of 0.28 nm correspond to the (332) planes of Na_15_Sn_4_. Additionally, the SAED pattern identifies the crystallographic planes of (130) for Na_9_Sn_4_ and (332) for Na_15_Sn_4_. This is in accordance with the XRD results. The compositionally modulated unsaturated Na_x_Sn_y_ alloys (x/y ≤ 15/4) are proposed to serve as an electrochemical buffer layer, which simultaneously optimizes both the thermodynamics and kinetics of ion diffusion. Based on the displacement reaction between Na metal and Sn ions, this strategy is highly versatile and can be extended to other alkali metal systems (e.g. Li, K) or alternative alloy phases (e.g. Na–Ga, Na–Sb) (Fig. S14).

### Behavior of Na plating and stripping

We hypothesize that the upper gradient alloy phase serves as an ion-buffering interlayer that synergistically regulates thermodynamic driving forces and ion-transfer kinetics to achieve dendrite-free morphology. In detail, the unsaturated Na–Sn alloy phases present in the gradient structure, such as the amorphous Na_x_Sn_y_ (x/y < 9/4), provide abundant sites for Na⁺ storage. During the Na plating process, these amorphous phases may transform into crystalline Na_9_Sn_4_, amorphous Na_3_Sn and crystalline Na_15_Sn_4_ phases, with Na_15_Sn_4_ and Na_9_Sn_4_ detectable via XRD characterization. Meanwhile, the gradient structure creates a built-in electric field that helps guide Na⁺ migration from the top to the bottom of the alloy, which simultaneously optimizes both the thermodynamics and kinetics of ion diffusion, thereby achieving dendrite-free morphology. In the subsequent stripping process, the built-in electric field (oriented opposite to ion migration) induces preferential Na⁺ extraction from the upper gradient alloy phase prior to the underlying metallic Na layer and the underlying metallic Na layer functions as an ion reservoir dynamically compensating for Na⁺ to maintain the structural stability of the gradient phase and mitigate the consumption of Na during long-term cycling. The driving force for the underlying sodium to be relayed and supplemented into the Na–Sn alloy can be explained from a thermodynamic perspective. The alloying process between Na and Sn is governed by the Gibbs free energy change, where the exothermic nature of the reaction and the increased configurational entropy from atomic mixing collectively result in the Gibbs free energy change being significantly lower. This energetic preference drives the spontaneous incorporation of Na⁺ ions to receive electrons and form the Na–Sn alloy. To validate our hypothesis regarding the Na plating and stripping behavior induced by the gradient alloy structure, the evolution of the crystal structure of the GNS/Na electrode was investigated using quasi-*in situ* XRD.

**Figure 3.**
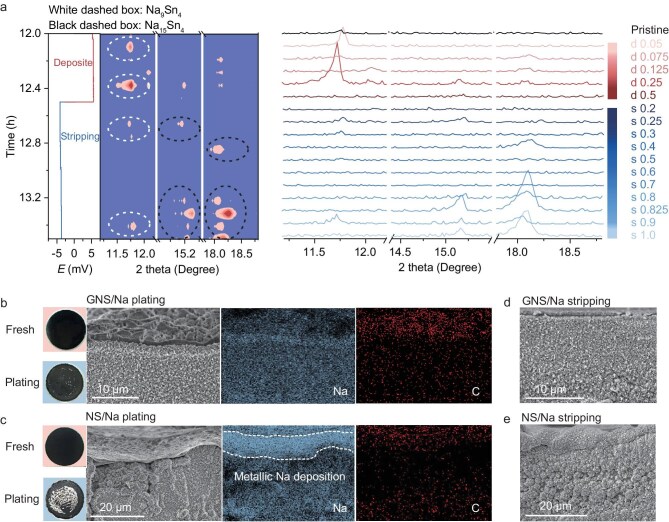
Structure and morphology evolution of gradient electrode after Na plating and stripping. (a) Quasi-*in situ* XRD patterns and data of GNS/Na with different Na plating and stripping. Optical and SEM images of the GNS/Na (b) and NS/Na (c) electrode after Na plating with capacity of 1 mAh cm^−2^. EDS mappings of Na and C elements are also provided. SEM images of GNS/Na (d) and NS/Na (e) electrodes after Na stripping with capacity of 1 mAh cm^−2^.

The presence of the Be window during quasi-*in situ* XRD testing may limit detection to the top of the sample, as it both weakens the X-ray signal and occupies a certain height, preventing detection throughout the entire sample. Consequently, alloy phases such as Na_9_Sn_4_ and Na_15_Sn_4_, which may exist in the middle and bottom regions of the gradient Na–Sn alloy structure, cannot be detected. Therefore, as shown in Fig. 3a, in the pristine GNS/Na, unsaturated Na_x_Sn_y_ (x/y < 9/4) alloy phases may be present at the top of the gradient alloy structure, but they are difficult to detect due to their amorphous nature. As a result, the pristine sample shows no relevant alloy peaks in the XRD pattern. The GNS/Na||Na asymmetric cell was assembled using pristine GNS/Na as the anode and pure Na as the cathode. The cell was first charged, as shown by the red curve in the charge–discharge profile. Correspondingly, the XRD patterns at different deposition amounts during charging are also indicated in red. After an initial deposition of 0.05 mAh cm^−2^, a sharp diffraction peak appears at 11.7°, characteristic of the Na_9_Sn_4_ alloy phase, indicating the transformation of amorphous unsaturated alloy phases [Na_x_Sn_y_ (x/y < 9/4)] into the Na_9_Sn_4_ phase upon Na alloying. However, with continued deposition up to 0.075 and 0.125 mAh cm^−2^, the 11.7° diffraction peak disappears, possibly indicating a further transition to the amorphous Na_3_Sn phase. Upon further deposition to 0.25 mAh cm^−2^, the intensity of the 11.7° peak increases significantly, suggesting that most of the Na_x_Sn_y_ (x/y < 9/4) has transformed into Na_9_Sn_4_. Continued deposition up to 0.5 mAh cm^−2^, the peak of 11.7° disappears again, indicating that Na_9_Sn_4_ may have undergone a transformation into amorphous Na_3_Sn phase. The above findings indicate the presence of abundant unsaturated Na–Sn alloy phases in the modified alloy structure, which provide ample sites for Na storage.

After the deposition of 0.5 mAh cm^−2^ of Na, the subsequent stripping process is initiated, as shown by the blue discharge curve and XRD patterns. After stripping 0.2 mAh cm^−2^ of Na, no alloy-related peaks are observed in the XRD pattern, suggesting that the alloy phases are not completely reversibly desodiated. When stripping continues to 0.25 mAh cm^−2^, weak peaks appear at 11.7° and 15.2°, corresponding to Na_9_Sn_4_ and Na_15_Sn_4_, respectively. This suggests that after the Na^+^ are transferred from the bottom Na foil, the upper amorphous Na_x_Sn_y_ (x/y < 9/4) and Na_3_Sn phases of the Na–Sn alloy structure may partially alloy to form Na_9_Sn_4_ and Na_15_Sn_4_, indicating that the built-in electric field (oriented opposite to ion migration) induces preferential Na⁺ extraction from the upper gradient alloy phase prior to the underlying metallic Na layer, and the underlying metallic Na layer functions as an ion reservoir dynamically compensating for Na⁺ to maintain the structural stability of the gradient phase. When stripping continues to 0.3 mAh cm^−2^, the alloy peaks disappear, indicating the Na^+^ extraction from Na_15_Sn_4_. As stripping continues to 0.4 mAh cm^−2^, a peak at 18.1° appears, also attributed to Na_15_Sn_4_, but disappears when stripping reaches 0.5 mAh cm^−2^. This validates our hypothesis. To further verify this conclusion, we continued the stripping process. After stripping to 0.6 and 0.7 mAh cm^−2^, no alloy peaks were detected. However, at 0.8 mAh cm^−2^, weak peaks attributed to Na_15_Sn_4_ appeared at 15.2° and 18.1°, which significantly intensified at 0.825 mAh cm^−2^. As stripping progressed to 0.9 mAh cm^−2^, the peak intensities decreased, and a new peak at 11.7°, corresponding to Na_9_Sn_4_, emerged. Notably, at 1.0 mAh cm^−2^ stripping, the Na_9_Sn_4_ peak at 11.7° disappeared, while the Na_15_Sn_4_ peaks at 15.2° and 18.1° reappeared. This observation further verifies the conclusion that the bulk Na reservoir ensures both the stability of gradient structure and compensation of Na^+^ depletion through dynamic ion replenishment during extended cycling.

Following the quasi-*in situ* XRD analysis of alloy phase changes during Na plating and stripping, optical and scanning electron microscopy (SEM) images provide further insights into the morphological evolution of the electrode. Figure 3b shows the SEM and optical images of the GNS/Na electrode after plating 1.0 mAh cm^−2^ of Na metal. The optical image shows almost no Na deposition on the surface of the GNS/Na electrode, while the SEM image reveals a dense alloy layer composed of uniformly sized particles, indicating that the unsaturated Na_x_Sn_y_ alloys thermodynamically suppress dendrite nucleation, and the potential gradients across the alloy phase kinetically regulate Na⁺ diffusion and deposition. Meanwhile, a thin NaEtO film overlaid on the alloy layer, being electron-insulating but ion-conductive, suppresses Na metal deposition on the surface. Energy dispersive X-ray spectroscopy (EDS) analysis indicates a similar Na content and higher C content in the NaEtO film compared to the underlying alloy layer. In contrast, the optical image in Fig. 3c shows substantial Na deposition on the NS/Na surface, while the SEM image shows that the alloy layer appears porous and loosely packed with larger particles in the upper region due to the inadequate ionic transport kinetics within the alloy phase, which induces persistent Na accumulation and volume expansion. This spatial redistribution generates a reverse electric field that impedes directional ion migration. Consequently, the surface of NS/Na is covered by a layer of Na metal, which EDS mapping shows to have a higher Na content and slightly higher C content compared to the underlying modified structure. The high-magnification SEM image shows an approximately 10 μm Na metal layer (Fig. S15), resulting from electric field inversion that promotes Na dendrite formation on the alloy surface. Additionally, the slightly higher C content in this layer compared to the underlying alloy is attributed to SEI formation from the decomposition of the organic electrolyte. Upon subsequent stripping of 1 mAh cm⁻^2^ of Na metal, the GNS/Na electrode maintains its morphology, without alloy phase fragmentation. This stability arises because the underlying metallic Na layer functions as a dynamic ion reservoir, maintaining the structural stability of the gradient alloy phase (Fig. 3d). In contrast, the NS/Na electrode exhibits a rough and porous morphology with non-uniform alloy particle distribution, likely due to the irreversible stripping failure of surface Na metal forcing Na^+^ extraction from the alloy phase, and thus triggering its mechanical fracture (Fig. 3e). Therefore, in the GNS/Na electrode, the compositionally modulated unsaturated Na_x_Sn_y_ alloys (x/y ≤ 15/4) within the upper gradient alloy phase serve as an electrochemical buffer layer, which simultaneously optimizes both the thermodynamics and kinetics of ion diffusion, thereby achieving dendrite-free morphology. Beneath this gradient layer, the bulk Na reservoir ensures both the stability of gradient structure and compensation of Na^+^ depletion through dynamic ion replenishment, overcoming the degradation issues plaguing NS/Na.

### Electrochemical performance

**Figure 4.**
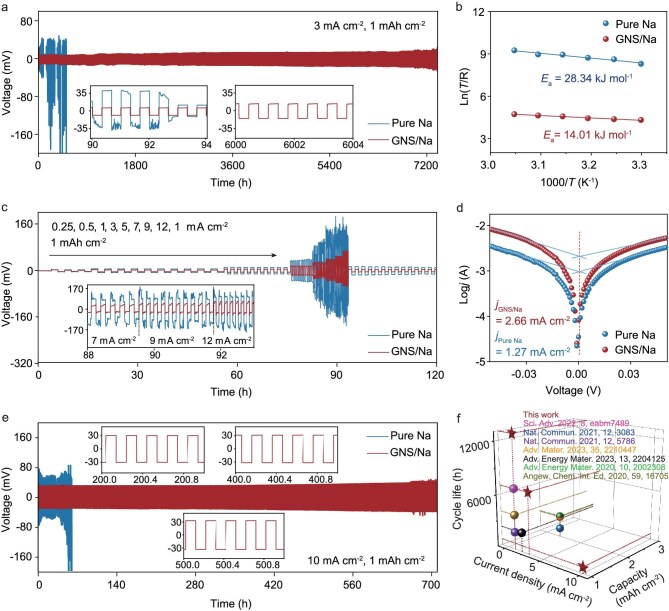
Electrochemical performance of the symmetric cells. (a) The voltage profiles of Pure Na and GNS/Na at a current density of 3 mA cm^−2^ and capacity of 1 mAh cm^−2^. (b) Rate performance of the Pure Na and GNS/Na electrodes. (c) The voltage profiles of GNS/Na at a current density of 10 mA cm^−2^ and capacity of 1 mAh cm^−2^. (d) Activation energy of Na^+^ diffusion through the SEI. (e) Exchange current density of the Pure Na and GNS/Na electrodes. (f) Comparation of symmetric cells of recently reported Na batteries in ether-based electrolytes.

The symmetric cell is one basic configuration to evaluate the anode stability and dendrite suppression capacity. Pure Na||Pure Na and GNS/Na||GNS/Na cells were assembled and tested at 1 and 3 mA cm^−2^ (1 mAh cm^−2^) (Fig. S16, Fig. 4a). The voltage–time graph shows the huge performance difference between Pure Na and GNS/Na. The GNS/Na||GNS/Na cell exhibits ultralong cycling stability for more than 12 000 h at 1 mA cm^−2^, and 7000 h at 3 mA cm^−2^, benefitting from the effective suppression of Na dendrite. Notably, the GNS/Na||GNS/Na cell at 3 mA cm^−2^ also exhibits low voltage hysteresis (18 mV after 6000 h of cycling), due to the built-in electric field that decreases the Na nucleation barrier, resulting in a lower deposition potential. In contrast, the Pure Na||Pure Na cell demonstrates a short cycle life of 92 h and suffers from a sharp increase in overpotential due to the accumulation of dendrites and a broken SEI. The insets are magnified views of the voltage curves. Pure Na shows sharp voltage fluctuations with an abrupt reduction in voltage at 92 h. Impressively, GNS/Na displays a smooth and flat voltage curve during cycling, suggesting homogeneous and stable Na deposition within the GNS/Na electrode. Moreover, when the plating/stripping capacity is increased to 3 mAh cm^−2^, GNS/Na maintains a stable overpotential of 10 mV and cycles for 750 h at a current density of 3 mA cm^−2^ (Fig. S17), indicating that the abundant unsaturated Na–Sn alloy phases within the GNS/Na can effectively store Na^+^. To evaluate the structural advantages for fast electrochemical kinetics, rate characterization of the symmetric cell at 1 mAh cm^−2^ was carried out, as shown in Fig. 4b. Apparently, compared to the Pure Na symmetric cell with a drastic voltage fluctuation at 5 mA cm^−2^ and high overpotential at 12 mA cm^−2^ (184 mV), GNS/Na can stably operate at increasing current density from 0.25 to 12 mA cm^−2^ (65 mV at 12 mA cm^−2^) and can recover stable cycling when the current is reduced to 1 mA cm^−2^, demonstrating the remarkable Na^+^ transfer kinetics of GNS/Na. The poor cycle stability of the Pure Na symmetric cell indicates that the SEI is unstable, leading to a large polarization. Therefore, in order to meet the needs of fast charging and discharging, the symmetric cell was tested at a larger current density of 10 mA cm^−2^. Encouragingly, the GNS/Na||GNS/Na cell can cycle for more than 700 h with a low potential of 25 mV, confirming the improvement of Na^+^ migration in the gradient alloy structure (Fig. 4c). To further prove the accelerated kinetics, the temperature-dependent electrochemical impedance spectroscopy (EIS) tests of symmetric cells were carried out to obtain the activation energy (E_a_) (Fig. 4d). The activation energy of the charge transfer by fitting to the Arrhenius equation is 14.01 kJ mol^−1^ in GNS/Na, which is lower than the 28.34 kJ mol^−1^ of the Pure Na, implying a much smaller activation barrier for the Na^+^ passing through the modified artificial structure. It means that the diffusion of Na^+^ is highly promoted by the gradient alloy structure. Furthermore, in the Tafel plots (Fig. 4e), there is a higher exchange current density of 2.66 mA cm^−2^ in GNS/Na compared to that of 1.27 mA cm^−2^ in NS/Na, indicating faster kinetics of the Na^+^ plating/stripping process with the help of the built-in electric field derived from the gradient alloy structure. Compared with studies reported in other top journals, which also involve ether-based electrolytes in Na batteries, the stability of our assembled GNS/Na||GNS/Na symmetric cell shows comparable and even superior performance at the same or a similar current density, as shown in Fig. 4f and Table S1 [16,17,22–32].

### Physicochemical characterizations of GNS/Na after cycling

**Figure 5.**
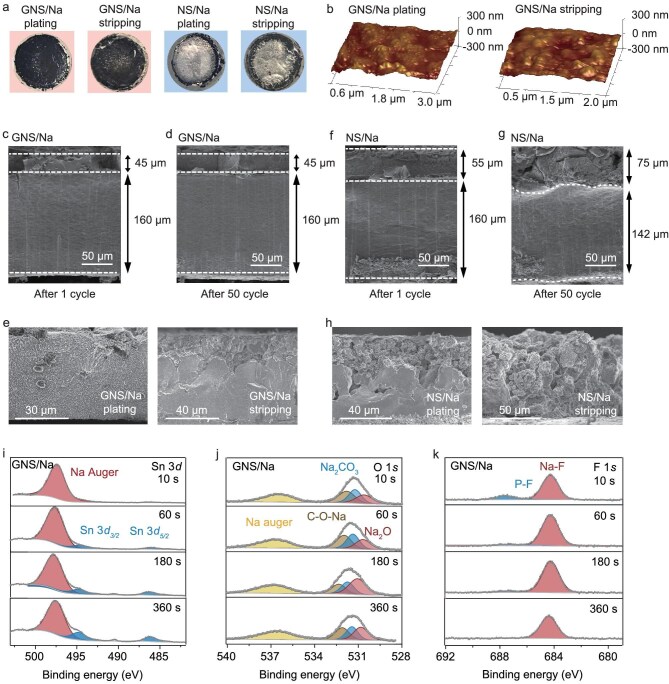
Micromorphology and SEI properties of electrodes after cycling. (a) Optical images of GNS/Na and NS/Na electrodes after Na plating and stripping. (b) 3D height maps of GNS/Na electrode after Na plating and stripping. Cross-sectional SEM images showing thickness and morphological evolution of GNS/Na electrode after 1 cycle (c) and 50 cycles (d) of plating. (e) SEM images of GNS layer of GNS/Na electrode after Na plating and stripping. Cross-sectional SEM images showing thickness and morphological evolution of NS/Na electrode after 1 cycle (f) and 50 cycles (g) of plating. (h) SEM images of NS layer of NS/Na electrode after Na plating and stripping. Sn 3*d* (i), O 1*s* (j) and F 1*s* (k) XPS spectra of GNS/Na electrode.

To evaluate the plating/stripping cycling stability of the GNS/Na electrode under harsh conditions, optical images were obtained after 50 cycles and SEM images were obtained after 1 cycle and 50 cycles at a current density of 10 mA cm^−2^ and areal capacity of 1 mAh cm^−2^ in the symmetric cell. Normally, 1 mAh cm^−2^ of Na deposition corresponds to a height increase of approximately 8.9 μm cm^−2^ on the plating side. Such a high areal capacity significantly amplifies interfacial reactions, surface fluctuations and dendrite growth over cycling. Impressively, the optical images reveal that neither the plating nor the stripping of the GNS/Na electrode exhibit noticeable Na deposition on the surface, whereas the NS/Na electrode displays significant Na metal accumulation (Fig. 5a). Atomic force microscopy (AFM) was applied to investigate the height maps of GNS/Na over cycling. The GNS/Na exhibits a smooth and uniform surface on both the plating and stripping sides (Fig. 5b), which is attributed to the upper gradient alloy phase serving as an ion-buffering interlayer that synergistically regulates thermodynamic driving forces and ion-transfer kinetics. In the SEM images of the GNS/Na anode, during the first cycle of Na plating, the unsaturated Na–Sn alloy in the top layer provides abundant Na^+^ storage sites. This alloying reaction leads to volume expansion, consequently increasing the alloy layer thickness from 40 µm (pristine) to 45 µm (Fig. 5c). After 50 cycles, the top alloy layer maintains both its thickness (Fig. 5d) and morphology (Fig. 5e) without alloy phase fragmentation, and the thickness of the underlying Na metal layer remains essentially unchanged. This indicates that the upper gradient alloy phase serves as an electrochemical buffer layer, simultaneously optimizing the thermodynamics and kinetics of Na^+^ diffusion, thereby preserving the overall structural stability of the anode.

In sharp contrast, for the NS/Na anode, after the first cycle of Na plating, the top alloy layer thickness increases from 40 to 55 µm, with a noticeable Na metal layer deposited on the surface (Fig. 5f). Morphologically, the alloy layer appears porous and loosely packed, with larger particles in the upper region. After 50 cycles, the top alloy layer thickness further increases to 75 µm (Fig. 5g), exhibiting a porous and fragmented morphology (Fig. 5h), while the underlying Na metal thickness decreases from 160 to 142 µm. This degradation stems from inadequate ionic transport kinetics within the alloy phase, leading to persistent Na accumulation in the upper region, which induces a reverse electric field that impedes directional Na^+^ migration and promotes Na dendrite formation on the alloy surface. During subsequent stripping cycles, the irreversible stripping failure of surface Na metal forces Na^+^ extraction from the alloy phase, and thus triggers its mechanical fracture. Concurrently, the Na^+^ released from the underlying metallic Na layer will migrate through the interparticle gaps to replenish the Na loss in the cathode, but they cannot be effectively replenished into the alloy layer to stabilize its structure. Consequently, these factors destroy electrode stability and cause significant morphological changes. XPS depth profile was also employed to study the species and longitudinal distribution of components in the SEI on the cycled GNS/Na electrode. After cycles in symmetric cells, the stripping-side electrodes were removed and cleaned with diglyme solvent. After Ar^+^ etching for 10 s, no Sn signal is observed; with the increase of sputtering time to 60 s, signals attributed to metallic Sn appear (485.81 and 494.61 eV) and intensify with continued etching for 180 and 360 s. It indicates that the surface of GNS/Na contains a thin SEI rather than Na metal deposition on the Na–Sn alloy structure (Fig. 5i). The O 1*s* spectra of GNS/Na can be fitted with three peaks. Peaks at 530.76, 531.26 and 532.01 eV are attributed to Na_2_O [33], Na_2_CO_3_ and C−O−Na [34], respectively (Fig. 5j). Na_2_O and Na_2_CO_3_ result from partial oxidation, while C−O−Na corresponds to RCH_2_ONa. Notably, the C−O−Na peak intensity remains unchanged throughout the etching process, indicating the persistence of the NaEtO in protecting the GNS/Na, which is electron-insulating but ion-conductive, thus preventing Na metal deposition. F 1*s* spectra of GNS/Na reveal two F-containing species after Ar^+^ etching for 10 s: Na−F (684.25 eV) and P−F (687.7 eV) [35], which could be credited to the decomposition of NaPF_6_ during cycling (Fig. 5k). It was observed that a P–F signal is only present after etching for 10 s, with no P–F signal detected for longer etching times. In contrast, the Na–F peak maintains a consistently high intensity after Ar^+^ etching for 10, 60, 180 and 360 s, suggesting the formation of a NaF-rich SEI. This is in agreement with previous studies indicating that Na–Sn alloys facilitate the formation of a NaF-rich SEI, which effectively stabilizes the electrode/electrolyte interface [15]. Furthermore, the strong adhesion of the organic P–F component contributes to mitigating the fracture of the rigid and brittle inorganic NaF-rich SEI. The upper gradient alloy phase serves as an ion-buffering interlayer that synergistically regulates thermodynamic driving forces and ion-transfer kinetics to achieve dendrite-free morphology, while also modulating SEI composition.

### Full cell performance

**Figure 6.**
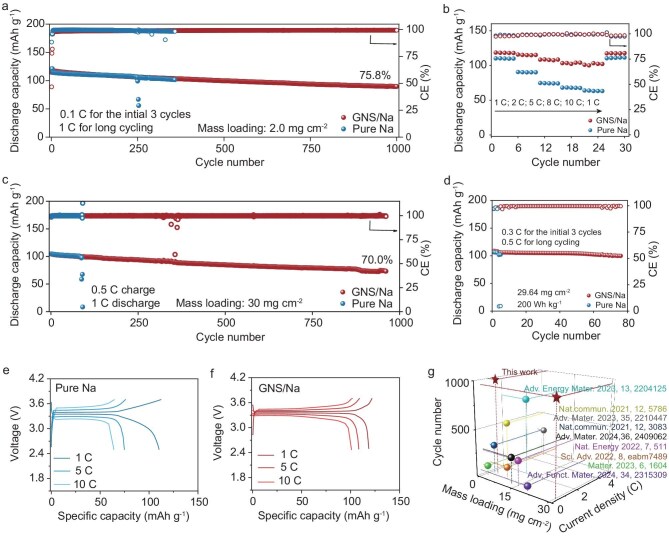
Electrochemical test of GNS/Na||NVP and Pure Na||NVP. Long-term cycling performances (a) and rate performances (b) of GNS/Na||NVP and Pure Na||NVP cells. Mass loading of NVP is 2.0 mg cm^−2^, with lean electrolyte. (c) Long-term cycling performances of GNS/Na||NVP and Pure Na||NVP cells. Mass loading of NVP is 30 mg cm^−2^, with flooded electrolyte. (d) Long-term cycling performance of GNS/Na||NVP cell. Mass loading of NVP is 30 mg cm^−2^, with lean electrolyte. Voltage profiles of the Pure Na||NVP (e) and GNS/Na||NVP (f) cells at various current rates. (g) Comparison of full cells of recently reported Na batteries in ether-based electrolytes.

To investigate the practical application of GNS/Na, the full cells were assembled with NVP as cathode to assess the feasibility and compatibility. As expected, the GNS/Na||NVP full cell displays superior cyclic stability and capability compared to the pure Na||NVP full cell. The full cells were operated at 1 C in the potential window of 2.5–3.7 V and the active NVP loading is 2.0 mg cm^−2^ with lean electrolyte (30 μL) (Fig. 6a). The GNS/Na||NVP full cell presents high-capacity retention and cycling stability, with a capacity retention of 75.8% and a Coulombic efficiency (CE) of 99.4% after 1000 cycles, while the pure Na||NVP full cell fails after 358 cycles with obvious CE decay. Moreover, the rate performance of the GNS/Na||NVP full cell also presents a higher capacity under different current densities than the pure Na||NVP full cell (Fig. 6b), indicating the significant improvement of Na^+^ diffusion kinetics. Additionally, we tested the cycling performance of GNS/Na||NVP cells under high and low temperature conditions. The results indicate that even under harsh temperature conditions, GNS/Na provides high capacities of 115.5 mAh g^−1^ at 60°C (Fig. S18a) and 95.7 mAh g^−1^ at −20°C (Fig. S18b). To verify the applications feasibility of the GNS/Na electrode, we assembled the GNS/Na||NVP pouch cell, which demonstrated a long cycle life of 100 cycles at a rate of 2 C (Fig. S19). The full cell coupled with a higher mass loading of NVP (30 mg cm^−2^) (Fig. 6c) cathode was also assembled and tested to further assess the performance under the high-capacity conditions. Notably, with a flooded electrolyte (100 μL), the GNS/Νa||NVP full cell manifests extremely stability and brilliant discharge capacity retention (from 104.5 to 73.2 mAh g^−1^; 70%) even after more than 950 cycles, in stark contrast to the pure Na||NVP full cell, which only discharges for 100 cycles. Furthermore, when using a thin GNS/Na anode, high mass-loading NVP cathode (∼2.92 mAh cm^−2^) and limited electrolyte (17 μL) [negative/positive areal capacity ratio (N/P) ∼0.77, electrode to cathode capacity ratio (E/C) ∼4.45 g Ah^−1^), the GNS/Na||NVP full cell can provide a high specific capacity of 109.0 mAh g^−1^ with a capacity retention rate of 92.6% after 79 cycles [charge/discharge at a current density of 58.5 mA g^−1^ (0.5 C) after activation at 0.1 C for 3 cycles], while the Pure Na||NVP cell can only undergo charge/discharge for 3 cycles at 0.1 C, and rapidly fails when the current density is increased to 0.5 C (Fig. 6d). The calculated GNS/Na||NVP coin cell (without packaging materials), with a high energy density of 200 Wh kg^−1^ (Table S2), reaches the advanced level in the field, which further confirms its practical properties. The rate performance of the GNS/Na||NVP full cell also presents lower polarization under different current densities than the pure Na||NVP full cell (Fig. 6e and f), indicating the significant improvement of Na^+^ diffusion kinetics. The EIS plots of the GNS/Νa||NVP and Pure Νa||NVP after different numbers of cycles are shown in Figs S20 and S21. Both the charge transfer resistance (R_ct_) and cathode electrolyte interphase resistance (R_CEI_) of the GNS/Νa||NVP cells are smaller than that of Pure Νa||NVP after different numbers of cycles (Table S3), indicating that the internal electric field in GNS/Na facilitates rapid Na^+^ migration, thereby reducing both charge transfer and diffusion impedance. These remarkable results surpass those of previous studies on Na batteries with ether-based electrolytes (Fig. 6g and Table S1) [16,17,22,24–26,31,36–38], particularly the few articles that have successfully tested full cells with cathodes at such a high mass loading (over 30 mg cm^−^^2^).

## CONCLUSION

We propose a hierarchically structured anode design that synergistically integrates thermodynamic stabilization, kinetic modulation and dynamic compensation to concurrently suppress dendrite formation and mitigate irreversible Na loss. This multi-scale strategy is realized through a spatially graded Na–Sn alloy/Na architecture with three functions: (i) the unsaturated alloy phases thermodynamically suppress dendrite nucleation; (ii) the potential gradients across the alloy layers kinetically control Na^+^ diffusion and deposition; and (iii) the bulk Na reservoir dynamically offsets irreversible Na loss, overcoming the cumulative degradation plaguing conventional anodes. As a result, the GNS/Na gradient electrode demonstrates exceptional performance, maintaining stable cycling for over 700 h at a high current density of 10 mA cm^−2^ and achieving a long cycle life of nearly 1000 cycles in a full cell under an ultra-high mass loading (30 mg cm^−2^). This strategy is highly versatile and can be extended to other materials and structures. Such architectures pave the way for sustainable, high-energy batteries compatible with industrial processing and renewable energy grid demands.

## METHODS

Detailed preparation and characterization methods for materials are available in the online Supplementary data.

## Supplementary Material

nwaf427_Supplemental_File

## References

[1] Li X, Li Z, Li C et al. Facilitating uniform lithium-ion transport via polymer-assisted formation of unique interfaces to achieve a stable 4.7 V Li metal battery. Natl Sci Rev 2025; 12: nwaf182. 40475066 10.1093/nsr/nwaf182 PMC12139002

[2] He J, Bhargav A, Su L et al. Tuning the solvation structure with salts for stable sodium-metal batteries. Nat Energy 2024; 9: 446–56. 10.1038/s41560-024-01469-y

[3] Tang Z, Zhang R, Wang H et al. Revealing the closed pore formation of waste wood-derived hard carbon for advanced sodium-ion battery. Nat Commun 2023; 14: 6024. 10.1038/s41467-023-39637-5 37758706 PMC10533848

[4] Lu Z, Yang H, Guo Y et al. Consummating ion desolvation in hard carbon anodes for reversible sodium storage. Nat Commun 2024; 15: 3497. 10.1038/s41467-024-47522-y 38664385 PMC11045730

[5] Li Y, Vasileiadis A, Zhou Q et al. Origin of fast charging in hard carbon anodes. Nat Energy 2024; 9: 134–42. 10.1038/s41560-023-01414-5

[6] Boebinger MG, Yarema O, Yarema M et al. Spontaneous and reversible hollowing of alloy anode nanocrystals for stable battery cycling. Nat Nanotechnol 2020; 15: 475–81. 10.1038/s41565-020-0690-9 32483321

[7] Xu F, Qu C, Lu Q et al. Atomic Sn–enabled high-utilization, large-capacity, and long-life Na anode. Sci Adv 2022; 8: eabm7489. 10.1126/sciadv.abm7489 35544572 PMC9094655

[8] Wei S, Xu S, Agrawral A et al. A stable room-temperature sodium–sulfur battery. Nat Commun 2016; 7: 11722. 10.1038/ncomms11722 27277345 PMC4906167

[9] Wang C, Sun Z, Liu Y et al. A weakly coordinating-intervention strategy for modulating Na^+^ solvation sheathes and constructing robust interphase in sodium-metal batteries. Nat Commun 2024; 15: 6292. 10.1038/s41467-024-50751-w 39060294 PMC11282164

[10] Choudhury S, Wei S, Ozhabes Y et al. Designing solid-liquid interphases for sodium batteries. Nat Commun 2017; 8: 898. 10.1038/s41467-017-00742-x 29026067 PMC5638817

[11] Fuchs T, Ortmann T, Becker J et al. Imaging the microstructure of lithium and sodium metal in anode-free solid-state batteries using electron backscatter diffraction. Nat Mater 2024; 23: 1678–85. 10.1038/s41563-024-02006-8 39313556 PMC11599044

[12] Tu Z, Choudhury S, Zachman MJ et al. Fast ion transport at solid–solid interfaces in hybrid battery anodes. Nat Energy 2018; 3: 310–6. 10.1038/s41560-018-0096-1

[13] Wang L, Ren N, Jiang W et al. Tailoring Na^+^ solvation environment and electrode-electrolyte interphases with Sn(OTf)_2_ additive in non-flammable phosphate electrolytes towards safe and efficient Na-S batteries. Angew Chem Int Ed 2024; 63: e202320060. 10.1002/anie.202320060 38285010

[14] Yang Z, Jiang H, Li X et al. Fabricating wide-temperature-range quasi-solid sodium batteries with fast ion transport via tin additives. Adv Funct Mater 2024; 34: 2407713. 10.1002/adfm.202407713

[15] Liu P, Miao L, Sun Z et al. Sodiophilic substrate induces NaF-rich solid electrolyte interface for dendrite-free sodium metal anode. Adv Mater 2024; 36: 2406058. 10.1002/adma.202406058 39097944

[16] Xu Y, Wang C, Matios E et al. Sodium deposition with a controlled location and orientation for dendrite-free sodium metal batteries. Adv Energy Mater 2020; 10: 2002308. 10.1002/aenm.202002308

[17] Jin Q, Lu H, Zhang Z et al. Synergistic manipulation of Na^+^ flux and surface-preferred effect enabling high-areal-capacity and dendrite-free sodium metal battery. Adv Sci 2022; 9: 2103845. 10.1002/advs.202103845 PMC8895136 35001541

[18] Crouch-Baker S, Deublein G, Tsai HC et al. Materials considerations related to sodium-based rechargeable cells for use above room temperature. Solid State Ionics 1990; 42: 109–15. 10.1016/0167-2738(90)90266-T

[19] Chevrier VL, Ceder G. Challenges for Na-ion negative electrodes. J Electrochem Soc 2011; 158: A1011. 10.1149/1.3607983

[20] Liu Y-R, Lei Z-W, Liu R-P et al. Sn-doped induced stable 1T-WSe_2_ nanosheets entrenched on N-doped carbon with extraordinary half/full sodium/potassium storage performance. Rare Met 2023; 42: 1557–69. 10.1007/s12598-022-02174-z

[21] Li X, Zhang X, Niu X et al. Strain retarding in multilayered hierarchical Sn-doped Sb nanoarray for durable sodium storage. Adv Funct Mater 2023; 33: 2300914. 10.1002/adfm.202300914

[22] Xu Z, Yang J, Zhang T et al. Stable Na metal anode enabled by a reinforced multistructural SEI layer. Adv Funct Mater 2019; 29: 1901924. 10.1002/adfm.201901924

[23] Wang Y, Dong H, Katyal N et al. A sodium–antimony–telluride intermetallic allows sodium-metal cycling at 100% depth of discharge and as an anode-free metal battery. Adv Mater 2022; 34: 2106005. 10.1002/adma.202106005 34679207

[24] Moorthy M, Moorthy B, Ganesan BK et al. A series of hybrid multifunctional interfaces as artificial SEI layer for realizing dendrite free, and long-life sodium metal anodes. Adv Funct Mater 2023; 33: 2300135. 10.1002/adfm.202300135

[25] Wang C, Zheng Y, Chen Z-N et al. Robust anode-free sodium metal batteries enabled by artificial sodium formate interface. Adv Energy Mater 2023; 13: 2204125. 10.1002/aenm.202204125

[26] Yue Q, Shen Z, Shi R et al. Na^+^-enriched quinoid polymer layer with fast ion transport for dendrite-free sodium metal batteries with high cyclic stability. ACS Energy Lett 2024; 9: 2265–75. 10.1021/acsenergylett.4c00376

[27] He X, Jin S, Miao L et al. A 3D hydroxylated MXene/carbon nanotubes composite as a scaffold for dendrite-free sodium-metal electrodes. Angew Chem Int Ed 2020; 59: 16705–11. 10.1002/anie.202006783 32530502

[28] Ye W, Li X, Zhang B et al. Superfast mass transport of Na/K via mesochannels for dendrite-free metal batteries. Adv Mater 2023; 35: 2210447. 10.1002/adma.202210447 36656991

[29] Yue L, Qi Y, Niu Y et al. Low-barrier, dendrite-free, and stable Na plating/stripping enabled by gradient sodiophilic carbon skeleton. Adv Energy Mater 2021; 11: 2102497. 10.1002/aenm.202102497

[30] Wang P, Zhang G, Wei X-Y et al. Bioselective synthesis of a porous carbon collector for high-performance sodium-metal anodes. J Am Chem Soc 2021; 143: 3280–3. 10.1021/jacs.0c12098 33645987

[31] Hou Z, Gao Y, Tan H et al. Realizing high-power and high-capacity zinc/sodium metal anodes through interfacial chemistry regulation. Nat Commun 2021; 12: 3083. 10.1038/s41467-021-23352-0 34035276 PMC8149847

[32] Qin J, Shi H, Huang K et al. Achieving stable Na metal cycling via polydopamine/multilayer graphene coating of a polypropylene separator. Nat Commun 2021; 12: 5786. 10.1038/s41467-021-26032-1 34599165 PMC8486844

[33] Zhao T, Zheng X, Wang D et al. A quasi-solid-state polyether electrolyte for low-temperature sodium metal batteries. Adv Funct Mater 2023; 33: 2304928. 10.1002/adfm.202304928

[34] Yan Y, Liu Z, Wan T et al. Bioinspired design of Na-ion conduction channels in covalent organic frameworks for quasi-solid-state sodium batteries. Nat Commun 2023; 14: 3066. 10.1038/s41467-023-38822-w 37244894 PMC10224921

[35] Zhu C, Wu D, Wang Z et al. Optimizing NaF-rich solid electrolyte interphase for stabilizing sodium metal batteries by electrolyte additive. Adv Funct Mater 2024; 34: 2214195. 10.1002/adfm.202214195

[36] Xie J, Li Z, Zheng X et al. Built-in electric field of in situ formed artificial interface layer induces fast and uniform sodium-ions transmission to achieve a long-term stable sodium metal battery under harsh conditions. Adv Funct Mater 2024; 34: 2315309. 10.1002/adfm.202315309

[37] Wang H, Wang J, Li W et al. Stable cycling of Na metal batteries at ultrahigh capacity. Adv Mater 2024; 36: 2409062. 10.1002/adma.202409062 39240064

[38] Yu L, He X, Peng B et al. Constructing ion diffusion highway in strongly coupled WSe_2_-carbon hybrids enables superior energy storage performance. Matter 2023; 6: 1604–21. 10.1016/j.matt.2023.03.013

