# Supplementary material for: Dynamic ion-buffering gradient bilayer anode realizes 200 Wh kg−1 dendrite-free sodium battery
Source: Natl Sci Rev. 2025 Sep 30;12(12):nwaf427. doi: 10.1093/nsr/nwaf427 (PMC12636514; doi:10.1093/nsr/nwaf427)
Supplement: nwaf427_Supplemental_File [file nwaf427_supplemental_file.pdf]

Dynamic Ion-Buffering Gradient Bilayer Anode realizes 200 Wh kg^{-1} Dendrite-free Sodium Battery

Siyang Ye, Shuanghui Han, Fei Tian, Danni Lei, Chengxin Wang**

MATERIALS

Na metal (⩾99.8%) was purchased from Sigma-Aldrich. Sn foil (99.9%) was purchased from Alfa Aesar. Cyclohexane (HPLC) and $Sn(EtO)_2$ were supplied by Macklin. Polypropylene membrane and glass fiber membrane (Whatman GF/A) were purchased from Celgard Co., Ltd. The commercial $Na_3V_2(PO_4)_3$ cathode (cathode loading of 12 mg cm^{-2} and 30 mg cm^{-2}, active material ratio is 92.6%) and power were bought from Guangdong Canrd New Energy Technology Co., Ltd. The electrolyte (1 M $NaPF_6$ in Diglyme) came from Suzhou Qianmin Chemical Reagent Company.

Preparation of The Na_9Sn_4 and $Na_{15}Sn_4$

The production details of the Na_9Sn_4 and $Na_{15}Sn_4$ are as follows. 1.247 g of tin foil and 0.966 g of Na metal were weighed and put in a corundum crucible. The crucible was then placed in a muffle furnace at 800°C for 2 minutes. After heating, the crucible was immediately removed, and the molten sample was cooling in argon to obtain $Na_{15}Sn_4$ alloy. To synthesize Na_9Sn_4, the procedure was identical, using 1.826 g of tin foil and 0.796 g of Na metal to obtain Na_9Sn_4 alloy. The $Na_{15}Sn_4$ alloy electrode was prepared by pressing 30 mg of $Na_{15}Sn_4$ powder into a 10 mm diameter pellet. The Na_9Sn_4 alloy electrode was prepared by pressing 30 mg of Na_9Sn_4 powder into a 10 mm diameter pellet. The $Na_9Sn_4/Na_{15}Sn_4$ alloy electrode was prepared by sequentially pressing 15 mg of $Na_{15}Sn_4$ powder and 15 mg of Na_9Sn_4 powder into a 10 mm diameter pellet.

Preparation of The GNS/Na and NS/Na

The production details of the GNS/Na and NS/Na are as follows. Na block was extruded into Na foil, then a cut-off knife was applied to it to prepare Na anodes with a diameter of 10 mm (for symmetric cells) and 14 mm (for full cells). 500 mg of $Sn(EtO)_2$ was dissolved in 50 mL of cyclohexane, then heated at 70°C and stirred at 1000 rpm for 7 days. After allowing the solution to stand, the supernatant was collected and designated as Solution 1. Separately, 100 mg of $Sn(EtO)_2$ was dissolved in 20 mL of cyclohexane, heated, and stirred under the same conditions for 5 days, and this was designated as

Solution 2. A mixture of 6 mL of Solution 1 and 0.4 mL of Solution 2 was then prepared, and 10 mm Na metal pieces were immersed in this mixture for 3 hours to obtain NS/Na. In this process, solution 1 is the clear supernatant obtained after standing, which contains a saturated solution of $Sn(EtO)_2$ dissolved in cyclohexane. Solution 2 is the turbid suspension containing the suspended $Sn(EtO)_2$ are homogeneously dispersed within the saturated solution. Due to the limited solubility of $Sn(EtO)_2$ in cyclohexane, the amount of $Sn(EtO)_2$ dissolved in solution 1 is insufficient to form a 40 μm alloy layer. Approximately 2 mg of additional $Sn(EtO)_2$ powder is required. However, directly weighing such small quantities (e.g., 2 mg) of $Sn(EtO)_2$ would introduce substantial relative errors. To address this, solutions 1 and 2 are strategically combined to achieve precise dosage control, thereby enhancing the reproducibility of the experiment. Finally, NS/Na was placed in a muffle furnace and heated at 110°C for 20 minutes with a heating rate of 5 °C per minute, then furnace-cooled to room temperature to obtain GNS/Na. The above operations are all done in an Argon filled glove box (Mikrouna, $H_2O < 0.1$ ppm, $O_2 < 0.1$ ppm).

Fabrication of The Cells

The NVP cathode with mass loading of 2.0 mg cm^{-2} was prepared by following steps. The mixture of NVP powder, acetylene black and polyvinylidene difluoride (PVDF, Solef 5130 by Solvay) with the ratio 7:2:1 by weight was manually grinded in an agate mortar for 30 mins and then dissolved in N-methyl-2-pyrrolidone (NMP, Sigma) by stirring for 4 h to form a slurry. The electrode slurry was then cast on an Al foil and dried at 80 °C for 12 h to remove the residue solvent. The NVP cathodes (diameter of 8 mm), matching Na_9Sn_4, $Na_{15}Sn_4$ and $Na_9Sn_4/Na_{15}Sn_4$ anode (diameter of 10 mm), and a separator (Celgard 2500 porous polypropylene film) were assembled into 2032 coin cells in an Argon filled glove box (Mikrouna, $H_2O < 0.01$ ppm, $O_2 < 0.01$ ppm). The amount of electrolyte injected into the full cells was 30 μL. The NVP cathodes (diameter of 12 mm), matching GNS/Na or Pure Na anode (diameter of 14 mm), and a separator were assembled into 2032 coin cells. The amount of electrolyte injected into the full cells was 30 μL. The symmetric cells (diameter of 10 mm) were assembled

using the same method with 30 μL of the electrolyte. Notably, when paired with a high-loading $Na_3V_2(PO_4)_3$ (30 mg cm^{-2}) cathode, the cell was tested with GF/A and PP separators wetted with 100 μL of electrolyte. When using thin GNS/Na anode, high mass loading NVP cathode (~2.92 mAh cm^{-2}), and limited electrolyte (17 μL) (N/P ≈ 0.77, E/C ≈ 4.45 g Ah^{-1}) was assembled into 2032 coin cells with PP separator. A pouch cell was fabricated with mass loading of approximately 2.0 mg cm^{-2} NVP cathode (length × width = 40 × 40 (mm/mm) and GNS/Na anode. The electrolyte was 1 M $NaPF_6$ in Diglyme.

Materials Characterization

X-ray diffraction (XRD) was performed on a D-MAX 2200 VPC X-ray diffractometer at a scan rate of 10° min^{-1}. Scanning electron microscopy (SEM, SU8010) with energy dispersive spectroscopy was used to characterize the morphology and elemental distribution. The composition was analyzed using transmission electron microscopy (TEM, JEOL/JEM-F200-TFEG). The surface chemical state of the anode was analyzed through X-ray photoelectron spectroscopy (XPS, ESCA Xi^+). Fourier-transform infrared spectroscopy (ATR-FTIR, Frontier, PerkinElmer) was also used with wavelength ranges from 500 to 4000 cm^{-1}. Atomic force microscope (AFM) and Kelvin probe force microscopy (KPFM) tests were performed using Bruker Dimension Icon. All air-sensitive samples were handled under strictly controlled conditions: SEM, XPS, and TEM samples were prepared and transferred using a vacuum-sealed apparatus, AFM and KPFM characterizations were conducted entirely within an argon-filled glovebox, while XRD samples were prepared in the glovebox and measured using airtight holders with Kapton film windows.

Electrochemical Characterizations

EIS measurements were performed on the full cells with mass loading of 2.0 mg cm^{-2}. The EIS was tested on DHMultiElec electrochemical workstation using the frequency range of 100 kHz to 0.1 Hz. To obtain the cycling and rate performance of the fabricated coin cells, the cells were placed in an incubator to maintain a constant operating

temperature of 30 °C and measured using a battery test system (LAND CT-2001A, Land Electronic Co., Ltd., Wuhan). Symmetric cells were cycled at specified current densities.

Supplementary Figures

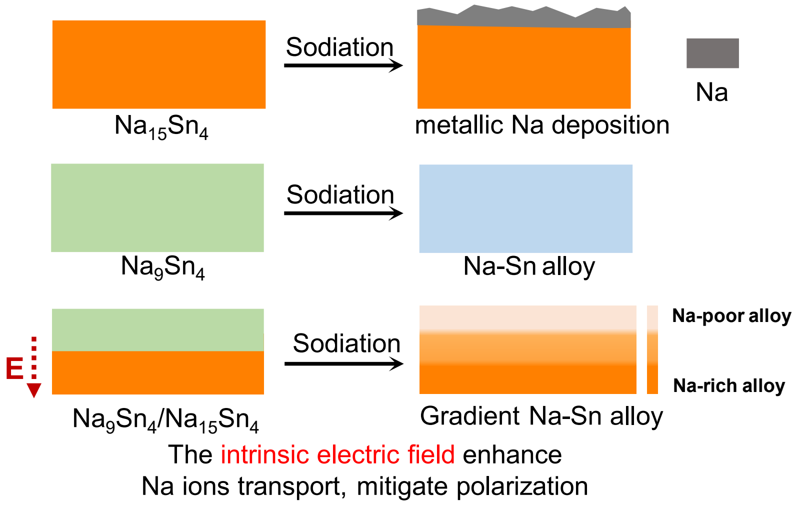

Supplementary Figure 1. Schematic illustration of the $Na_{15}Sn_4$, Na_9Sn_4 and $Na_9Sn_4/Na_{15}Sn_4$ (GNS) electrodes after sodiation.

To investigate the effects of an intrinsic electric field in a gradient Na-Sn alloy (GNS) electrode on Na ions transport and cycling stability, we selected Na_9Sn_4 and $Na_{15}Sn_4$ due to their distinct electrochemical potentials and sodium storage properties. Na_9Sn_4, with a higher potential (0.16 V vs. Na) compared to $Na_{15}Sn_4$ (0.08 V vs. Na)[1-2], functions as an unsaturated sodiated phase that can further alloy with Na ions. In contrast, $Na_{15}Sn_4$, being an almost fully sodiated phase, facilitates efficient Na ions transport but has limited capacity to further alloy with Na ions. By combining these two phases, we aim to construct a gradient alloy electrode with an intrinsic electric field, which is hypothesized to promote the migration of Na ions, accelerate the interfacial transport dynamics and reduce internal polarization of battery.

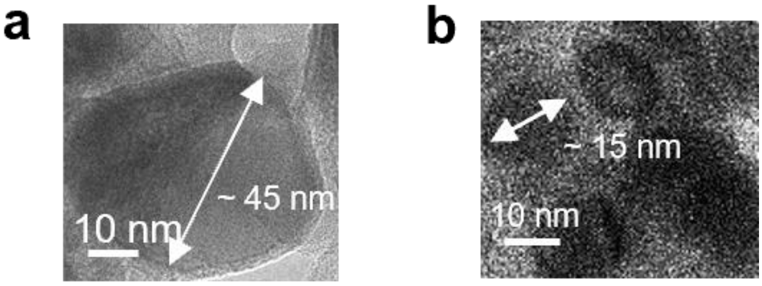

Supplementary Figure 2. High-resolution TEM images of $Na_{15}Sn_4$ (a) and Na_9Sn_4 (b).

We first prepared Na_9Sn_4 and $Na_{15}Sn_4$ alloys by high-temperature melting of Na and Sn in the appropriate proportions. The molten alloys were then cooled and ground to obtain Na_9Sn_4 and $Na_{15}Sn_4$ alloy powders. The morphology and size of the Na_9Sn_4 and $Na_{15}Sn_4$ alloy nanoparticles were investigated using transmission electron microscopy (TEM). The $Na_{15}Sn_4$ nanoparticles, with an average size of 45 nm (Supplementary Fig. 2a), are approximately three times larger than the Na_9Sn_4 nanoparticles (about 15 nm) (Supplementary Fig. 2b), which is consistent with the volume expansion observed in Na-Sn alloys after sodiation.

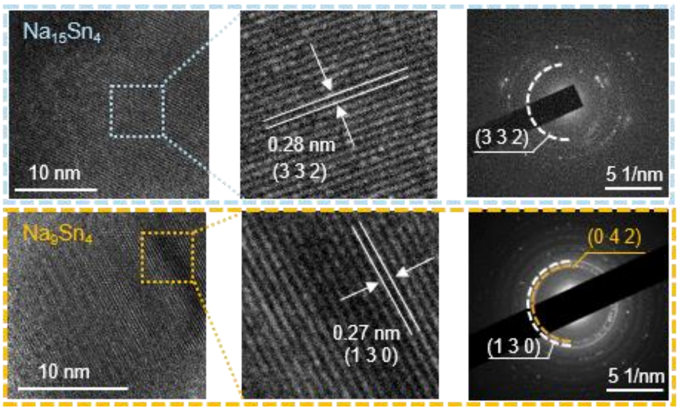

Supplementary Figure 3. High-resolution TEM images and selected-area electron diffraction of $Na_{15}Sn_4$ and Na_9Sn_4.

In the high-resolution TEM (HRTEM) image of $Na_{15}Sn_4$ alloy nanoparticles, the measured lattice fringes of 0.28 nm match well with the (3 3 2) crystal plane of $Na_{15}Sn_4$. The diffraction rings observed in the selected area electron diffraction (SAED) pattern provide additional confirmation of the nanocrystalline nature of the $Na_{15}Sn_4$ nanoparticles. Similarly, the observed lattice fringe of 0.27 nm aligns precisely with the (1 3 0) crystal plane of Na_9Sn_4, and the SAED pattern identified the corresponding crystallographic planes of (1 3 0) and (0 4 2).

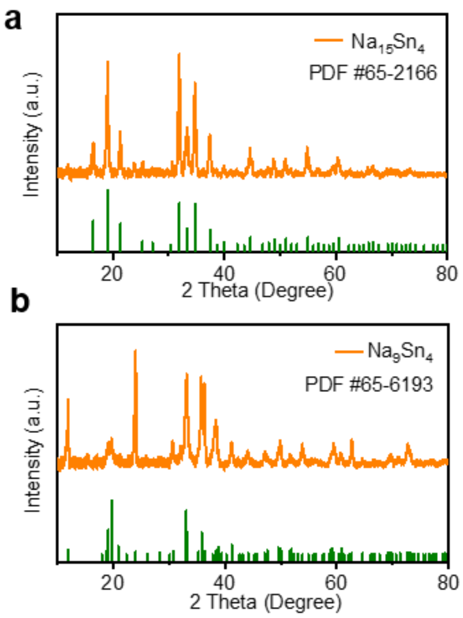

Supplementary Figure 4. XRD patterns of $Na_{15}Sn_4$ (**a**) and Na_9Sn_4 (**b**).

X-ray diffraction (XRD) analysis was employed to verify the successful synthesis of Na_9Sn_4 and $Na_{15}Sn_4$ alloys. The XRD patterns of both Na_9Sn_4 and $Na_{15}Sn_4$ samples exhibited no detectable signals corresponding to metallic Na and Sn, indicating the complete conversion of the initial metallic Na and Sn into the designated alloy phases. Specifically, the diffraction peaks (16.511°, 19.088°, 21.366°, 31.919°, 33.379°, 34.784° and 37.457°) corresponding to $Na_{15}Sn_4$ (JCPDS No. 65-2166, cubic) alloy phase can be simultaneously detected, and the relative intensities are consistent with the standard XRD patterns, confirming the successful synthesis of $Na_{15}Sn_4$ alloy (Supplementary Fig. 4a). Similarly, the diffraction peaks (11.942°, 19.127°, 19.824°, 24,015°, 33.218°, 35.817° and 38.815°) corresponding to Na_9Sn_4 alloy phase align with the standard XRD patterns (JCPDS No. 65-6193, orthorhombic) (Supplementary Fig. 4b). However, due to preferred orientation induced by cold grinding, the peaks at 11.942° and 24.015° exhibit higher intensities, while those at 19.824° and 19.127° show weaker intensities.

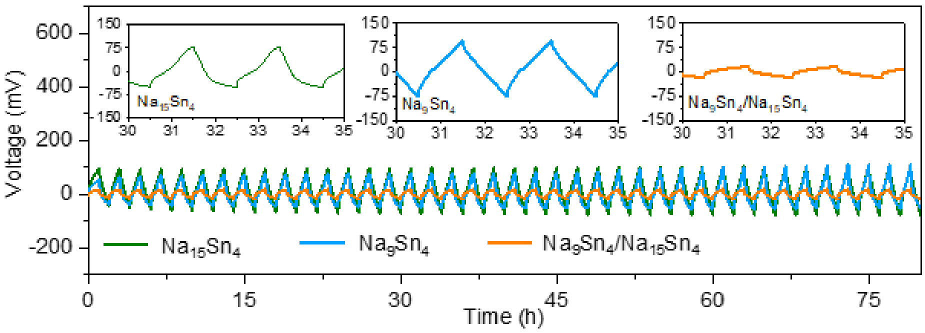

Supplementary Figure 5. Cycling performance of symmetric cells ($Na_{15}Sn_4||Na_{15}Sn_4$, $Na_9Sn_4||Na_9Sn_4$ and $Na_9Sn_4/Na_{15}Sn_4||Na_9Sn_4/Na_{15}Sn_4$).

To illustrate the advantages of the gradient design, pure-phase $Na_{15}Sn_4$ and Na_9Sn_4 alloy powders were initially pressed into pellets to construct $Na_{15}Sn_4||Na_{15}Sn_4$ and $Na_9Sn_4||Na_9Sn_4$ symmetric cells. Due to the potential differences between Na_9Sn_4 and $Na_{15}Sn_4$, Na_9Sn_4 and $Na_{15}Sn_4$ phases were pressed into two distinct layers in a 1:1 mass ratio to form $Na_9Sn_4/Na_{15}Sn_4$ electrode with an intrinsic electric field. Subsequently, $Na_9Sn_4/Na_{15}Sn_4||Na_9Sn_4/Na_{15}Sn_4$ symmetric cell was assembled. As shown in Supplementary Fig. 5, the $Na_9Sn_4/Na_{15}Sn_4||Na_9Sn_4/Na_{15}Sn_4$ symmetric cell consistently maintains the lowest overpotential of 20 mV during cycling, while the single-phase symmetric cells ($Na_{15}Sn_4||Na_{15}Sn_4$ and $Na_9Sn_4||Na_9Sn_4$) exhibit progressively higher overpotentials, exceeding 75 mV. This indicates that the intrinsic electric field within the gradient Na-Sn alloy electrode effectively promotes the migration of Na ions, accelerate the interfacial transport dynamics and reduce internal polarization of battery.

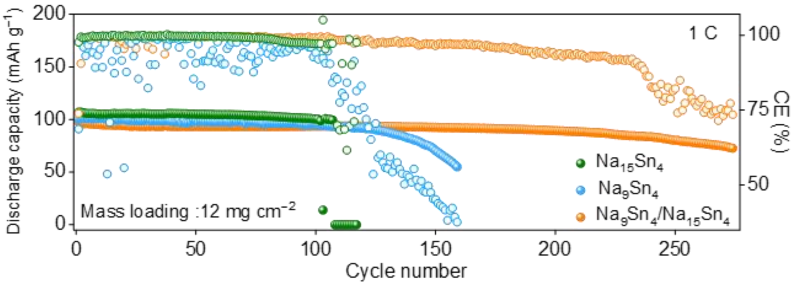

Supplementary Figure 6. Long-term cycling performances of full cells ($Na_{15}Sn_4$||NVP, Na_9Sn_4||NVP and $Na_9Sn_4/Na_{15}Sn_4$||NVP) under 1 C. Mass loading of NVP is approximately 12.0 mg cm^{-2}.

To evaluate the performance of the gradient Na-Sn alloy electrode in practical applications, we assembled the full cells paired with a high mass loading NVP cathodes (12 mg cm^{-2}) and compared the long-cycle stability. As shown in Supplementary Fig. 6, the $Na_{15}Sn_4$||NVP full cell exhibits relatively stable performance over 110 cycles, with a coulombic efficiency (CE) above 99% until after 80 cycles. However, after 100 cycles, the CE gradually decreases to 97.24%, and drops significantly to 68.35% after 110 cycles. This rapid decline in performance is attributed to the fact that $Na_{15}Sn_4$ is an almost fully sodiated phase, which lacks the capacity to accommodate additional Na storage. Consequently, Na ions deposit as metallic Na on the $Na_{15}Sn_4$ surface. The highly reactive Na metal undergoes side reactions with the organic electrolyte, forming the SEI that continuously consumes active Na ions during cycling. Efforts to compensate for the Na loss through dealloying induce $Na_{15}Sn_4$ particles fracture, which degrades the structural integrity and stability of the $Na_{15}Sn_4$ electrode. For the Na_9Sn_4 electrode, as an unsaturated Na-Sn alloy phase, although it can alloy with Na ions and prevent Na dendrite formation, the substantial volume expansion and shrinkage of the alloy during repeated sodiation/desodiation cycles at high areal capacities of 1 mAh cm^{-2} causes cracking and pulverization of the alloy particles. The freshly exposed alloy surface further accelerates electrolyte decomposition, generating a thick SEI that continuously consumes active Na. This results in poor discharge specific capacity and unstable CE during cycling. Consequently, the Na_9Sn_4||NVP full cell exhibits significant fluctuations in CE over 100 cycles and rapidly declines to 30% after 160 cycles. For the $Na_9Sn_4/Na_{15}Sn_4$||NVP full cell, the CE remains above 97% until after 150 cycles, which is superior to that of the control cell without intrinsic electric field.

However, after 200 cycles, the CE gradually declines to 93% and then decreases rapidly to 74% after 250 cycles. Although the intrinsic electric field within the gradient Na-Sn alloy structure can regulate Na ion transport dynamics and improve cycling stability to some extent, the inherent issues related to significant volume expansion/shrinkage during repeated sodiation/desodiation cycles, especially at high areal capacities, prevent it from maintaining long-cycle stability. Therefore, the Na-Sn alloy electrode is not suitable for high-capacity conditions due to its interfacial instability during cycles.

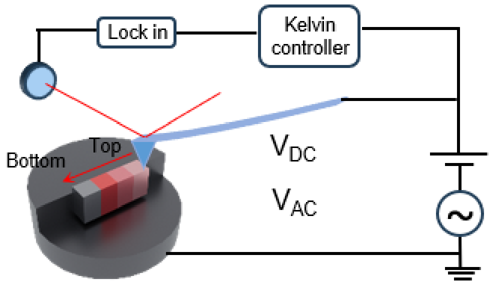

Supplementary Figure 7. Schematic illustration of KPFM setup.

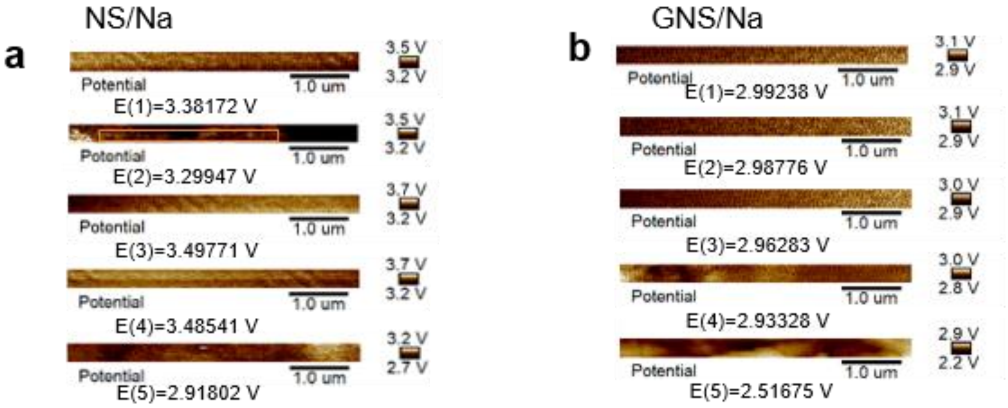

Supplementary Figure 8. Distribution map and image raw mean potential of NS/Na (a) and GNS/Na (b).

The potential of the GNS/Na is consistently lower than that of the NS/Na, reflecting the successful sodiation of Sn to form Na-Sn alloy phases through Na diffusion. Notably, the results showed no regular potential variation in the NS/Na modified structure (3.38172 V, 3.29947 V, 3.49771 V, 3.48541 V, 2.91802 V), whereas the GNS/Na layer exhibited a gradual decrease in potential from the surface to the substrate (2.99238 V, 2.98776 V, 2.96283 V, 2.93328 V, 2.51675 V), confirming the successful construction of the internal electric field within the GNS/Na electrode.

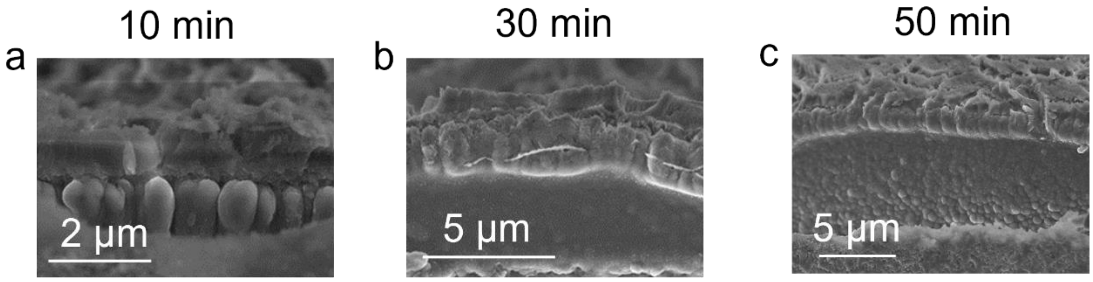

Supplementary Figure 9. (a-c) Cross-sectional SEM image of Na anode after different time of soaking.

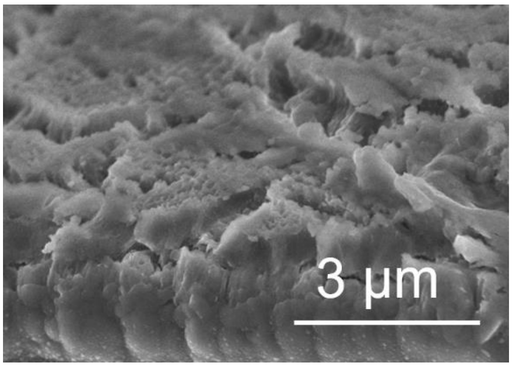

Supplementary Figure 10. Cross-sectional SEM image of NS/Na electrode surface after 3 hours reaction.

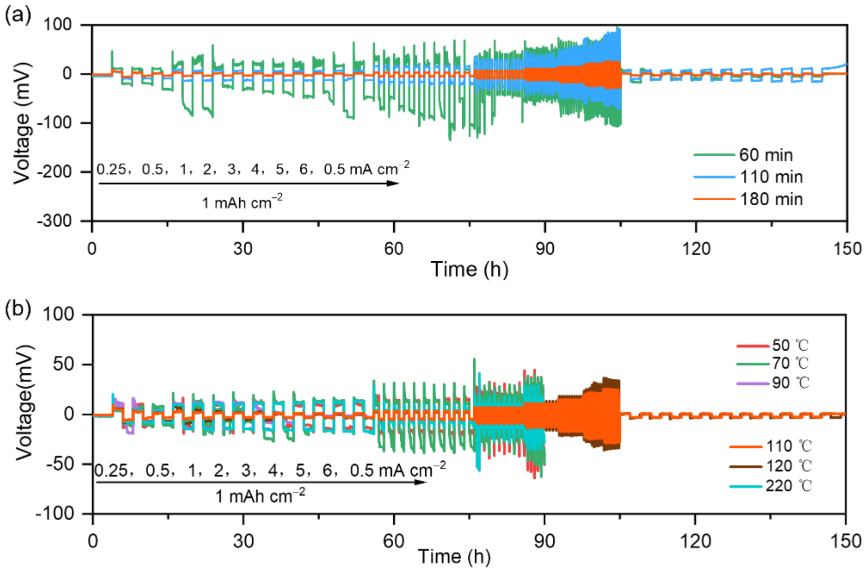

Supplementary Figure 11. (a) Galvanostatic voltage profiles of GNS/Na after Na soaking for different time. The samples were placed in a muffle furnace, heated at 110°C for 20 minutes with a heating rate of 5 °C min^{-1}, and subsequently furnace-cooled to room temperature. (b) Galvanostatic voltage profiles of GNS/Na after heated for different temperatures. The samples were heated at the corresponding target temperatures for 20 minutes with a heating rate of 5 °C min^{-1}, followed by furnace cooling to room temperature.

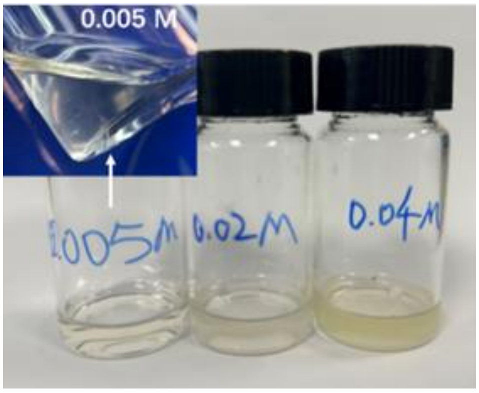

Supplementary Figure 12. Optical images of 1 M $NaPF_6$ in diglyme electrolytes containing different concentrations of NaOEt after 24 hours of stirring.

To determine the solubility of NaEtO, we prepared electrolytes by adding different concentrations of commercial NaEtO (0.005 M, 0.02 M, and 0.04 M) to the baseline electrolyte of 1 M $NaPF_6$ in diglyme (Supplementary Figure 12). After stirring for 24 h, the 0.02 M and 0.04 M samples remained turbid, indicating that NaEtO could not dissolve in the electrolyte. For the 0.005 M sample, a small amount of undissolved NaEtO was still observed after stirring. These results strongly suggest that NaEtO has extremely low solubility in the electrolyte.

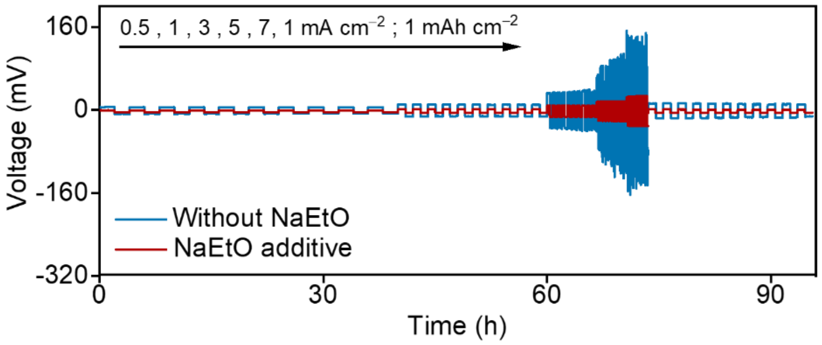

Supplementary Figure 13. Galvanostatic voltage profiles of symmetric Na||Na cells with and without 0.005 M NaEtO additive in 1 M $NaPF_6$/diglyme electrolyte.

To further evaluate whether the trace amount of dissolved NaEtO would affect battery performance, we assembled symmetric Na||Na cells using the baseline electrolyte with and without 0.005 M NaEtO additive. As shown in Supplementary Figure 13, the cells containing NaEtO consistently exhibited lower polarization voltages at all tested current densities and maintained stable operation even at 7 mA cm^{-2}, whereas the NaEtO-free cells showed continuously increasing polarization at 5 mA cm^{-2}.

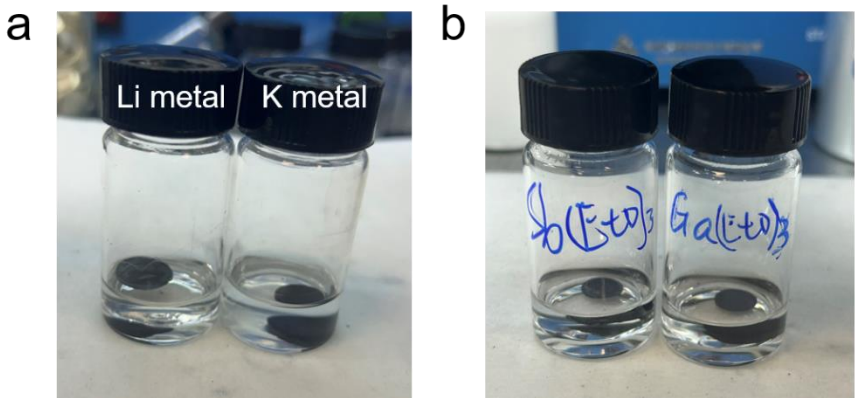

Supplementary Figure 14. (a) Li and K metal plates were immersed in cyclohexane solutions containing $Sn(EtO)_2$. (b) Na metal plate was immersed in cyclohexane solutions containing $Sb(EtO)_3$ and $Ga(EtO)_3$.

According to the design principle of our work, which relies on the displacement reaction between Na metal and Sn ions, it is theoretically feasible to extend this strategy beyond the Na-Sn system. We immersed Li and K metal plates in cyclohexane solutions containing $Sn(EtO)_2$, and in both cases, similar phenomena to those observed in the Na-Sn system occurred (Supplementary Figure 14a). In addition, we tested other metal alkoxides, including $Sb(EtO)_3$ and $Ga(EtO)_3$, by dissolving them in cyclohexane and immersing Na plates. In both cases, similar phenomena to those observed in the Na-Sn system occurred (Supplementary Figure 14b). These results indicate that our in-situ displacement strategy can indeed be extended to other alkali metal systems (e.g., Li, K) as well as to alternative alloy phases (e.g., Na-Sb, Na-Ga).

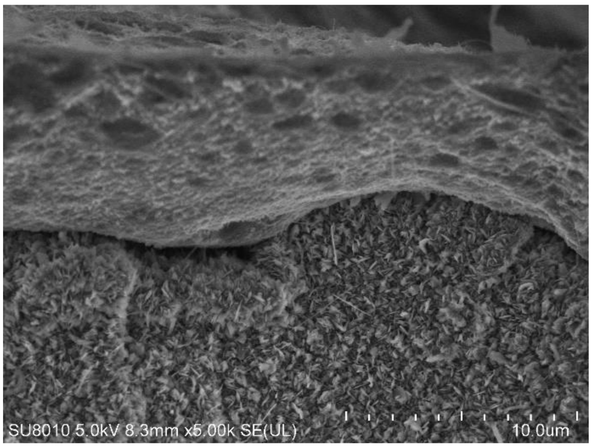

Supplementary Figure 15. Cross-sectional SEM image of NS/Na electrode after Na plating with capacity of 1 mAh cm^{-2}.

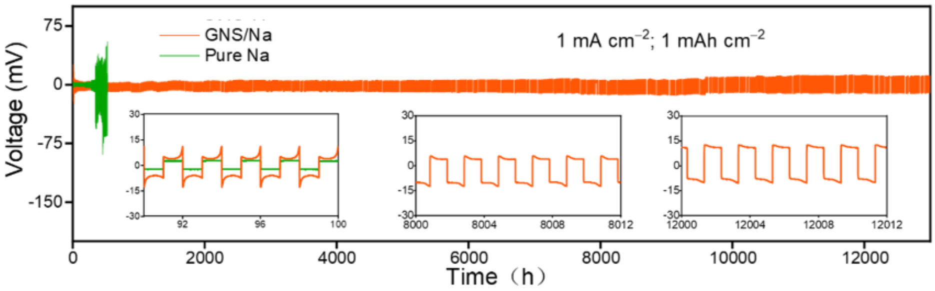

Supplementary Figure 16. Galvanostatic voltage profiles of GNS/Na and Pure Na symmetric cells at 1 mA cm^{-2}; 1 mAh cm^{-2}. Inset is the enlarged figure of the curves.

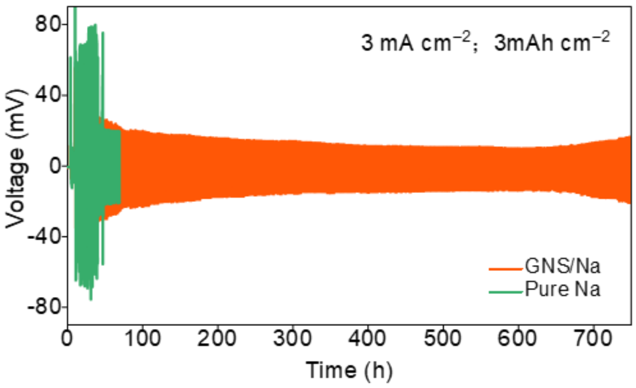

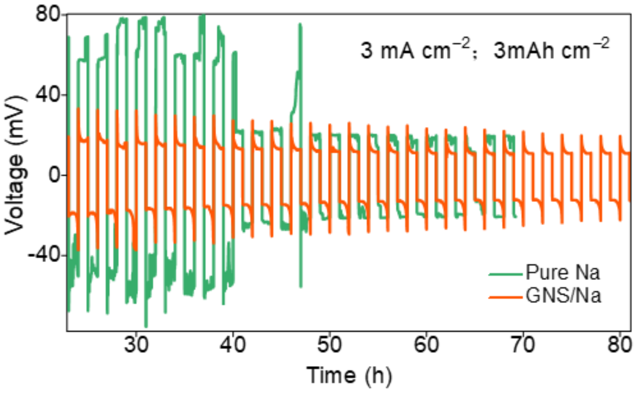

Supplementary Figure 17. (a) Galvanostatic voltage profiles of GNS/Na and Pure Na symmetric cells at 3 mA cm^{-2}; 3 mAh cm^{-2}. (b) Enlarged figure of the galvanostatic voltage profiles.

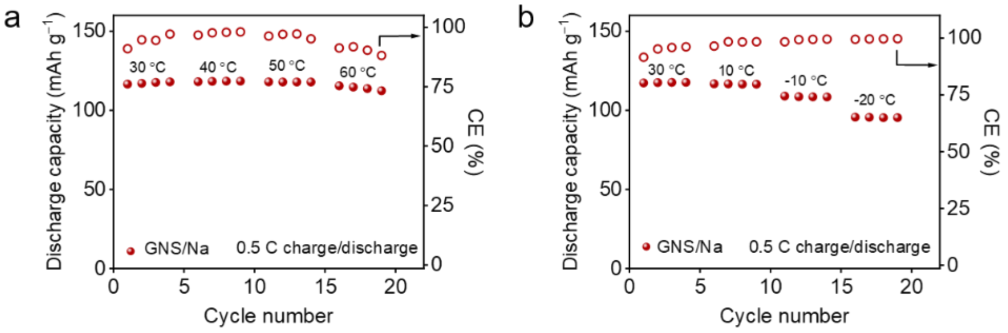

Supplementary Figure 18. Cycling performances of GNS/Na||NVP cells under high (a) and low (b) temperature conditions.

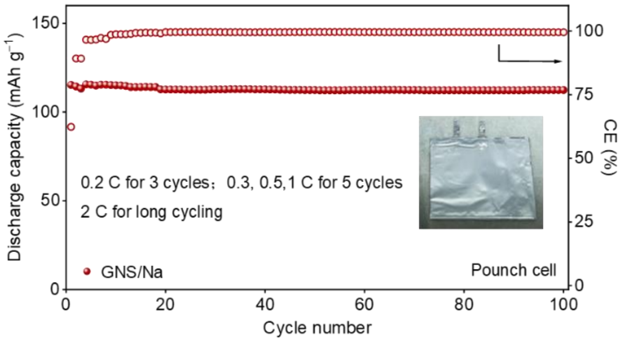

Supplementary Figure 19. Cycling performances of GNS/Na||NVP pouch cell.

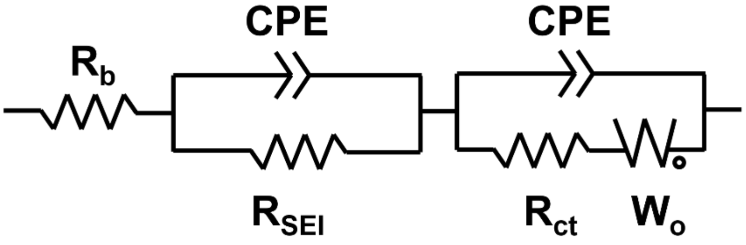

Supplementary Figure 20. Equivalent circuit model used for the EIS simulation.

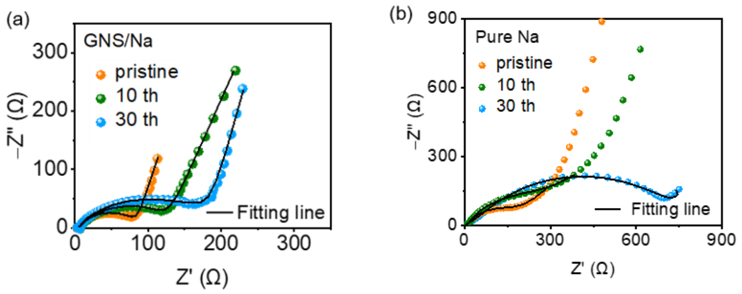

Supplementary Figure 21. EIS plots of GNS/Na||NVP cell (a) and Pure Na||NVP cell (b) after different cycles.

Supplementary Tables

Supplementary Table 1 Comparation of symmetric cells and full cells of recently reported sodium batteries in ether-based electrolytes.

Strategy classification	Samples	Symmetric cells			Full cells			Ref.
		Current density (mA cm^{-2})	Areal capacity (mAh cm^{-2})	Lifespan (h)	Cathode	Mass loading (mg cm^{-2})	Cycle number	
**Composite anode**	**Gradient NaSn-alloy/Na**	**3**	**1**	**7000**	**NVP**	**2.5**	**1000**	**This work**
		**1**	**1**	**13000**		**30**	**1000**	
**Anode-free**	Graphitic carbon-coated Al foil	——	——	——	$Na[Cu_{1/9}Ni_{2/9}Fe_{1/3}Mn_{1/3}]O_2$	15	260	*Nature Energy.* 2022, **7**, 511–519
**Separator modification**	Sn-coated separator	2	2	1000	NVPF	1	300	*Nat. Commun.* 2021, **12**, 3083
	mPG-coated separator	1	1	2000	NVP	1	500	*Nat. Commun.* 2021, **12**, 5786

**Anode design**	WSe_2-carbon anode	——	——	——	NVP	0.8-1.2	100	*Matter.* 2023, **6**, 1604
**3D host**	At-Sn @HCN/Na	1	1	7000	NVP	7	100	*Sci. Adv.* 2022, **8**, eabm7489
	PAN fiber coated with Sn	2	2	2500	NVP	4	600	*Adv. Energy Mater.* 2020, **10**, 2002308
	3D MXene/Carbon Nanotubes	1	1	4000	——	——	——	*Angew. Chem. Int. Ed.* 2020, **59**, 16705 – 16711
	Hollow open-mesochannels carbon spheres	2	2	2400	$Na_2Fe_xMn_{(1-x)}Fe(CN)_6$	2	300	*Adv. Mater.* 2023, **35**, 2210447
**Artificial SEI**	Na-In alloy/Na	2	2	790	NVP	10	100	*Adv. Funct. Mater.* 2019, **29**, 1901924
	HCOONa-Na	2	1	2200	NVP	10	800	*Adv. Energy Mater.* 2023, **13**, 2204125
	PDBM/Na	2	1	1300	NVP	3	900	*ACS Energy Lett.* 2024, **9**, 2265−2275

	Na/Ag	——	——	——	NVP	20	50	*Adv. Funct. Mater.* 2024, **34**, 2315309
**Interlayer**	CNT interlayer	1	1	9000	NVP	12	270	*Adv. Mater.* 2024, **36**, 2409062

Supplementary Table 2 Cell parameters of GNS/Na||NVP coin cell for 200 Wh kg^{-1}.

**Parameter**		**Assigned Value**
**Cathode areal capacity**		2.92 mAh cm^{-2}
**Anode areal capacity**		2.26 mAh cm^{-2}
**Active material ratio**		92.6 wt%
**N/P ratio**		0.77
**Discharge capacity**		108.95 mAh g^{-1}
**Average discharge voltage**		3.3560 V
**Assigned density**	NVP cathode	29.64 mg cm^{-2}
	Sodium metal anode	1.93 mg cm^{-2}
	Al foil	3.3 mg cm^{-2}
	Electrolyte	13.02 mg cm^{-2}
	Separator	1.03 mg cm^{-2}
	Total	48.92 mg cm^{-2}
**Specific energy**		200.32 Wh kg^{-1}

Supplementary Table 3 Simulation results of GNS/Na||NVP, Pure Na||NVP at different cycles.

	GNS/Na		Pure Na	
	R_{CEI}/Ω	R_{ct}/Ω	R_{CEI}/Ω	R_{ct}/Ω
0	1.322	81.48	5.483	183.4
10 th	4.198	122.9	7.341	337.9
30 th	1.686	158.6	33.45	747.1

Supplementary References

1. Crouch-Baker, S., Deublein, G., Tsai, H.C., Zhou, L.Z. & Huggins, R.A. Materials considerations related to sodium-based rechargeable cells for use above room temperature. Solid State Ionics 42, 109-115 (1990).

2. Chevrier, V.L. & Ceder, G. Challenges for Na-ion negative electrodes. Journal of The Electrochemical Society 158, A1011 (2011).
